# Effect of Residents' Previous Laparoscopic Surgery Experience on Initial Robotic Suturing Experience

**DOI:** 10.5402/2012/569456

**Published:** 2012-09-02

**Authors:** Gokhan Sami Kilic, Teresa M. Walsh, Mostafa Borahay, Burak Zeybek, Michael Wen, Daniel Breitkopf

**Affiliations:** ^1^Department of Obstetrics & Gynecology, The University of Texas Medical Branch, 301 University Blvd, Galveston, TX 77555-0587, USA; ^2^Department of Obstetrics and Gynecology, Ege University School of Medicine, Bornova, 35100 Izmir, Turkey; ^3^Department of of Obstetrics and Gynecology, The Mayo Clinic, 200 First Street SW, Rochester, MN 55905, USA

## Abstract

*Objective*. To assess the impact of gynecology residents' previous laparoscopic experience on the learning curve of robotic suturing techniques and the value of initial structured teaching in dry lab prior to surgery. *Methods*. Thirteen gynecology residents with no previous robotic surgery experience were divided into Group 1, consisting of residents with 2 or fewer laparoscopic experiences, and Group 2, consisting of residents with 3 or more laparoscopic experiences. Group 1 had a dry-laboratory training in suturing prior to their initial experience in the operating room. *Results*. For all residents, it took on average 382 ± 159 seconds for laparoscopic suturing and 326 ± 196 seconds for robotic suturing (*P* = 0.12). Residents in Group 1 had a lower mean suture time than residents in Group 2 for laparoscopic suturing (*P* = 0.009). The residents in Group 2, however, had a lower mean suture time on the robot compared to Group 1 (*P* = 0.5). *Conclusion*. Residents with previous laparoscopic suturing experience may gain more from a robotic surgery experience than those with limited laparoscopic surgery experience. In addition, dry lab training is more efficient than hands-on training in the initial phase of teaching for both laparoscopic and robotic suturing skills.

## 1. Introduction

Over the last 2 decades, advances in surgical technology combined with a growing consumer demand have created a shift towards gynecologic minimally invasive surgery (MIS). The safety and efficacy of MIS have been thoroughly documented in the literature and include decreased blood loss, a shorter hospital stay, faster recovery time, fewer wound-related complications, and superior cosmetic results [1, 2]. The da Vinci Surgical System (Intuitive Surgical, Sunnyvale, CA, USA) for use during gynecologic procedures has quickly become the minimally invasive alternative to conventional laparoscopy by providing surgeons with greater dexterity and improved visualization of the surgical field. Along these advantages, this new technology also raises new liability issues [3].

This shift towards MIS in gynecology has required that academic institutions train their residents and fellows in new technology while simultaneously teaching the fundamentals of open surgery. However, these expectations are becoming increasingly difficult in an environment of limited resident work hours and increased financial concerns where respecting patient safety and satisfaction is essential. As a result, a push has been made to discover the most effective way to train residents and fellows in both laparoscopic and robotic surgery. Becoming competent in MIS requires adequate preparation time to acquire this unique set of skills. The importance of preparation via MIS simulation labs prior to entry into the operating theater is well documented [4]. However, whether or not the skills gained from laparoscopy translate into improved robotic surgery skills for physicians in training is yet to be explored. Previous studies by surgeons investigating their personal experiences in learning robotic-assisted gynecologic surgery partly attributed their short learning curves to their extensive experience with advanced laparoscopic procedures prior to using robotic assistance [5–8]. While these previous studies suggest that surgeons with more laparoscopic experience learn robotic surgery more quickly, it is not yet known if that data can be extrapolated to resident physicians. Current data is limited on the impact of previous laparoscopic exposure on learning robotic surgery skills for physicians in training.

Laparoscopic suturing is arguably the hardest task while learning laparoscopic surgery because it requires a mastery of a complex set of maneuvers and skills. Although the increased dexterity provided by the robot has decreased the difficulty of suturing, it still represents one the fundamental skills that must be learned by trainees in robotic surgery. The aim of the study was to evaluate if previous experience with laparoscopic surgery among gynecology residents could influence—and perhaps shorten—the learning curve for robotic suturing.

## 2. Methods

 This study was approved by the Institutional Review Board at the University of Texas Medical Branch, Galveston TX, USA. A total of 13 residents were recruited for participation in this study: 4 third-year residents (PGY3) and 9 fourth-year residents (PGY4). None of the residents who participated in this study had any previous robotic surgery experience. All residents received a didactic lecture on MIS suturing. Third-year residents had an additional dry laboratory with hands-on training in suturing prior to their initial experience in the operating room. All but 2 of the PGY4s already had exposure to MIS suturing during their previous experience in the operating room, so they received no additional dry-lab teaching. The 2 PGY4s with very limited hands-on MIS experience received the same training as the PGY3s. Group 1 consisted of all PGY3s and the 2 PGY4s considered to be MIS-naive residents. The more MIS-experienced PGY4s, all of whom had at least 3 previous MIS experiences, made up Group 2.

 Both groups of residents were evaluated using 2 different techniques: robotic suturing with intracorporeal knot tying and laparoscopic suturing using extracorporeal knot tying. In both the laparoscopic and robotic approaches, the vaginal cuff was closed using a 0 polyglactin (Vicryl, Ethicon) on a CT-1 needle in an interrupted suture closure. All residents were timed for each suture placed. The suture time was defined as the elapsed time for loading the needle, placing 4 square knots, and cutting the suture.

### 2.1. Statistical Analysis

 A *t*-test compared mean suturing times by surgery modality and stratified by groups of clinicians. The Satterthwaite correction was applied when the test for heterogeneity of variances between groups was significant.

## 3. Results

**Figure 1:**
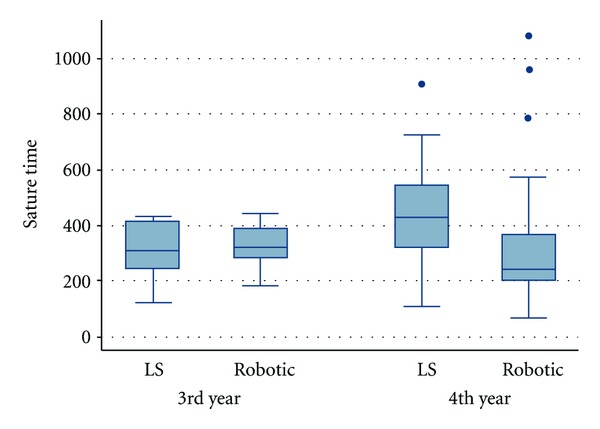
Comparison of laparoscopic and robotic suturing times in PGY3 versus PGY4.

**Figure 2:**
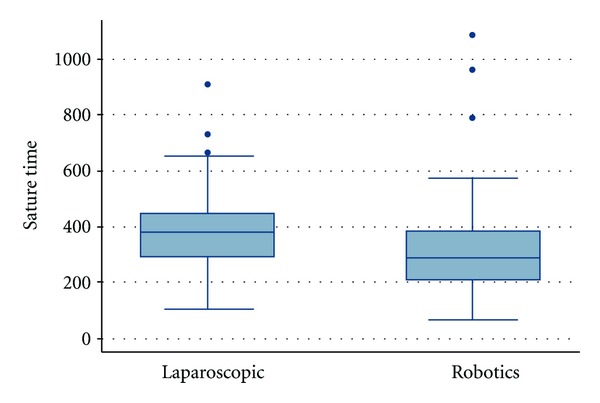
Comparison of mean robotic suturing time of all residents compared to the mean laparoscopic suturing time.

 The mean suturing times of the PGY3 and PGY4 residents are shown in Table 1. Table 1 demonstrates that PGY3s had a shorter suturing time than their PGY4 counterparts in both robotic and laparoscopic suturing. For the PGY3 group, no statistically significant difference (*P* = 0.5) was found between the mean suturing time in robotic suturing (235 ± 337 seconds) versus laparoscopic suturing (158 ± 457) (Table 1, Figure 1). The PGY4 group's mean suture time was 337 ± 235 seconds for robotic suturing and 457 ± 158 seconds for laparoscopic suturing, a statistically significant difference  (*P* = 0.02) (Table 1, Figure 1). For all residents, it took on average 382 ± 159 seconds for laparoscopic suturing and 326 ± 196 seconds for robotic suturing, which was not found to be statistically significant (*P* = 0.12) (Figure 2).

 The mean suturing times between PGY3 and PGY4 for combined robotic and laparoscopic suturing, was also compared (Table 2). The difference between the mean suture times in the PGY3 (310 ± 95.8 seconds) versus PGY4 (393 ± 210.6 seconds) groups was found to be statistically significant (*P* = 0.01) (Table 2).

 The surgical experience of the residents was also taken into account (Table 3). Residents in Group 1 (*n* = 6) had 2 or fewer previous laparoscopic surgery experiences, and those in Group 2 (*n* = 7) had 3 or more cases (average = 4.4) prior to enrollment in the study. The residents in Group 1 had a significantly lower mean suture time than those residents in Group 2 for laparoscopic suturing (*P* = 0.009). The residents in Group 2 had a lower mean suture time on the robot compared to Group 1; however, this difference did not achieve significance (*P* = 0.5).

## 4. Discussion

 In the literature, a variety of publications report about surgical education using robotic systems [9–11] and the learning curve for robotic-assisted surgery [12–14]. However, our study is the first to examine the effect that gynecology residents' previous laparoscopic experiences have on their initial experiences with robotically assisted suturing in the operating room. The results from our study demonstrate that previous laparoscopy experience improves the learning curve for robotic surgery. As shown in Table 1, a statistically significant difference between the means of the laparoscopic suturing time and the robotic suturing time occurred in the PGY4 group, which had more overall laparoscopic experience. The less-experienced PGY3s did well laparoscopically but did not show the same type of time reduction for robotic suturing. However, our findings also indicated the importance of structured teaching in dry labs prior to beginning in the operating room. Lee et al. described the robot as an “enabling technology” because it may allow those with less-advanced laparoscopic skills to perform minimal access procedures they would otherwise do via an open approach [15]. Our results support the idea that physicians in training must first learn the basics in a well-established dry-lab environment prior to relying on robot technology.

 The primary limitation of our study is that of sample size, as both study groups were composed of a small number of residents, likely affecting the power of the statistical analysis. We stopped enrolling participants in the study when the gynecology MIS department implemented the use of a braided suture for vaginal cuff closure. A second limitation of our study involves selection bias with regard to surgical case difficulty assigned to the different resident groups. Trials with larger number of trainees and using braided sutures to reflect the trend in closure are needed before coming to a definitive conclusion.

 In conclusion, introducing the robotically assisted suturing technique to our residents resulted in a steep learning curve regardless of their previous experience in laparoscopy. However, our data suggest that residents with previous laparoscopic-suturing experience will gain more from a robotic-surgery experience than those residents with minimal or no previous laparoscopic-surgery experience. Furthermore, initial introduction in a dry-lab setting decreases the learning curve for residency training. As suturing and knot tying are the bases of minimal access surgery, the need for specific training for all surgeons who aim to become competent with the techniques is paramount. In an era of increasing financial concerns and decreasing resident work hours, the need to effectively train residents in a time efficient manner, while keeping patients safe, is also a priority.

## Figures and Tables

**Table 1:** Comparison of mean suturing times in resident groups in laparoscopic and robotic surgery.

	Laparoscopy (mean ± SD sec)	Robot (mean ± SD sec)	*P* value
PGY3 (*n* = 4)	158 ± 457	235 ± 337	0.5
PGY4 (*n* = 9)	457 ± 158	337 ± 235	0.02
All residents (*n* = 13)	382 ± 159	326 ± 196	0.12

*P* < 0.05: statistically significant.

**Table 2:** Comparison of suturing time between PGY3 and PGY4 for both methods combined.

	PGY3 (mean ± SD sec)	PGY4 (mean ± SD sec)	*P* value
Laparoscopic and robotic suturing times combined	310 ± 96	393 ± 211	0.01

*P* < 0.05: statistically significant.

**Table 3:** Comparison of mean suturing times in resident groups in laparoscopic versus robotic surgery (robot) with different levels of experience.

	Group 1 (*n* = 6)	Group 2 (*n* = 7)	*P* value
Laparoscopy (mean sec)	309.76	417.88	0.009
Robot (mean sec)	348.14	291.28	0.5

*P* < 0.05: statistically significant.
